# Prevalence of mental disorders and work ability among unemployed individuals in Germany: a register-based analysis of socio-medical assessments by the Federal Employment Agency between 2016 and 2021

**DOI:** 10.1186/s12889-025-21603-z

**Published:** 2025-02-05

**Authors:** Patrik Roser, Kirsi Manz, Norbert Scherbaum, Gabriele Lotz-Metz, Andreas G. Franke

**Affiliations:** 1 Department of Psychiatry and Psychotherapy, Medical Faculty, LVR-University Hospital Essen, University of Duisburg-Essen, Virchowstraße 174, 45147 Essen, Germany; 2 Department of Adult Psychiatry and Psychotherapy, Medical Faculty, University Hospital of Psychiatry Zurich, University of Zurich, Zurich, Switzerland; 3 Department of Hematology, Hemostasis, Oncology and Stem Cell Transplantation, Hannover Medical School, Hannover, Germany; 4 Federal Employment Agency, Medical Services, Nuernberg, Germany; 5 University of Applied Labour Studies, Federal Employment Agency, Mannheim, Germany

**Keywords:** Unemployment, Work ability, Mental disorders, Epidemiology, Public health

## Abstract

**Background:**

The interactions between unemployment and mental health are complex. However, broad and current epidemiological data about the mental health status of unemployed individuals in Germany are scarce. This study aimed to evaluate the prevalence rates of mental disorders and work ability among all unemployed people who underwent socio-medical assessment by the Federal Employment Agency (FEA).

**Methods:**

Socio-medical assessments between 2016 and 2021 were taken from the FEA database and analyzed regarding sociodemographic characteristics, mental disorders and work ability. Standard descriptive statistics were used to analyze the data.

**Results:**

A total of 4,249,028 unemployed individuals were assessed. Of these, 2,213,048 persons (52.1%) had at least one psychiatric diagnosis (mean age 40.6 ± 13.5 years, 51.7% female). Mood disorders (53.9%), neurotic, stress-related and somatoform disorders (43.9%), as well as substance use disorders (15.3%) showed the highest prevalence rates among mental disorders and accounted for about 80% of all psychiatric diagnoses. About 40% of them were evaluated to be able to work full time.

**Conclusions:**

Psychiatric morbidity among unemployed people is high. However, a significant proportion of them was assessed to be able to return to the labor market. Therefore, close collaborations between unemployment agencies and mental health care institutions as well as specific re-integration programs including supported job placement and vocational training, long-term job coaching as well as integrated mental health care are required in order to improve mental health status, prevent further chronification, avoid labor market exit, and increase employment rate.

## Background

Mental disorders may negatively influence work performance and increase workplace absences, thereby increasing the risk of unemployment and poor socioeconomic status over the work-life course. The greatest risk of unemployment was reported for people suffering from schizophrenia, bipolar disorder, major depression, anxiety as well as alcohol and other substance use disorder [1–3]. According to a recent cross-sectional study from Germany, only 27.9% of patients with severe mental illness were working in the primary labor market, whereas 25.9% were unemployed and 23.3% had retired for mental health reasons [4]. Low psychosocial functioning as well as concurrent chronic somatic illness were identified as strong predictors of poor labor market outcome among people with severe mental disorders [5]. Once unemployed, people with mental disorders also have more difficulties in finding any employment compared to unemployed people without mental disorders, which cannot be attributed solely to symptoms and limited capacity for work but also to discrimination by employers [6].

On the other hand, unemployment has repeatedly been reported to have detrimental effects on mental health, not only at the time of job loss, but also in the long term [7]. In this regard, an extensive meta-analysis reported that the prevalence of psychological problems among unemployed people of 34% was more than 2-fold higher compared to 16% among employed individuals [8]. In comparison to different employment groups, including employed in full or part-time, mini-jobbers, house wives/men, workers in voluntary services, and older-age retired people, unemployed people had the highest rates of mental health problems and the worst psychological capacity profiles [9]. In particular, unemployed people have a higher risk for the development of depression, anxiety and psychosomatic symptoms, lower self-esteem, and poorer health-related quality of life [8, 10]. In this respect, male sex, younger age, previous blue-collar employment and duration of unemployment were identified as significant moderators of psychological distress among unemployed, whereas higher education level and social support appeared to attenuate the negative effects of unemployment on mental health [8, 11]. Moreover, longer duration of unemployment was related to greater risk of suicide and suicide attempt across almost all diagnosis groups [12]. Unemployment in combination with severe social crises, such as economic recessions or the COVID-19 pandemic, have an even stronger impact on psychological symptoms and mental well-being [13–14]. Conversely, return to work has been found to markedly improve mental health, particularly psychological distress and depression [15].

Possible mechanisms underlying the association between unemployment and poor mental health include the lack of the manifest functions of employment, i.e., financial benefits, and the so-called latent deprivation due to the lack of time-structure, social contacts, collective purpose, social status, and enforced activity, but also the stigmatization of people who are unemployed and/or mentally ill [16]. Besides psychological distress due to unemployment and its impact on mental health, unemployed people often show health risk behaviors, such as cigarette smoking and risky alcohol drinking, do less physical exercise, participate less in health prevention programs, experience more social exclusion, and show a higher rate of poverty compared to employed people; all of these factors may in turn have additional negative effects on mental well-being [17–20].

In Germany, about 6.0% (*N* = 2,790,529) of the population of working age is currently (October 2024) unemployed [21]. The Federal Employment Agency (FEA) is the principal authority for all labor market issues, such as evaluation of unemployment rates and all services to integrate unemployed in the labor market. Basic security benefits for jobseekers, so-called citizen’s benefits, for long-term unemployed people (> 1 year) in accordance with Book II of the German Social Law (SGB II) as well as employment promotion benefits in accordance with Book III (unemployment < 1 year) comprise benefits to ensure the means of subsistence and benefits for integration into employment, respectively. Registered jobseekers are obliged to regularly attend employment agencies in order to document job search activities, accept job offers or undergo socio-medical assessments, if appropriate. The assessments are carried out on request of FEA counselors to determine a person’s work ability and the extent to which clients can be integrated into the labor market [22]. The evaluation is performed by medical practitioners from the Department of Medical Services (MS). The FEA is responsible for about 500,000 socio-medical assessments per year, either with direct interaction containing clinical examination and, in the uppermost cases, in-depth medical history taking by requesting medical files from practitioners (e.g., family doctors, specialists, hospitals) based on a patient’s confidentiality release, or, in a minority of cases, without direct interaction according to records.

To date, broad and current epidemiological data about the mental health status of unemployed individuals are scarce. However, such data are fundamental to estimate specific supply needs and to provide recommendations for mental health care systems as well as employment-promoting programs. Therefore, the aim of this study was to systematically analyze all socio-medical assessment data of a predefined time period provided by the FEA, and to evaluate the prevalence of mental disorders as well as the work ability among the respective unemployed people.

## Methods

Anonymized register data of all unemployed clients who underwent socio-medical assessment between 2016 and 2021 by the MS was taken from the main data storage of the FEA in October 2023 for further analysis. Each data set comprised sociodemographic characteristics, such as age and gender, physical and mental health status, as given by the two main diagnoses according to ICD-10, as well as socio-medical assessment results which contain information on the continuous prospective work ability concerning skilled or unskilled work in the general labor market in three performance categories (< 3 h, 3–6 h, > 6 h per day). This quantitative capacity defines the time frame in which gainful employment is reasonable in relation to work intensity, work-related attitudes, and work organization [22]. For this study, only data of clients with at least one diagnosis of the ICD-10 Chapter V: Mental and behavioral disorders (F00-F99) was included.

Standard descriptive statistics were used to analyze the data. Continuous variables were summarized as mean and standard deviation (SD) and categorical variables were given as frequency and percentage. The differences between two or more continuous variables were assessed by using t-test and analysis of variance (ANOVA), respectively. The differences between two categorical variables were assessed by Fisher’s exact test. All statistical analyses were performed using the software R, version 4.3.2.

In compliance with the guidelines of the Federal Commissioner for Data Protection and Information Security (BfDI), the study data did not contain any personally identifiable information. The study was approved by the Data Protection Department of the FEA (Ref. No. AZ1400.12-1/2023).

## Results

Between 2016 and 2021, a total of 4,249,028 socio-medical assessments were carried out. Of these, 2,213,048 data sets (52.1%) of FEA clients with at least one psychiatric diagnosis were identified and considered for further analysis. Overall, a mental disorder as first diagnosis was coded in 87.7% and as second diagnosis in 64.3% of the clients. With regard to the sociodemographic characteristics, there were slightly more female (51.7%) than male (48.3%) clients with at least one psychiatric diagnosis. The mean age of the clients was 40.6 (± 13.5) years. Between 2016 and 2021, the percentage of female clients increased significantly from 51.1 to 51.9% (*p* < 0.001) and the mean age increased from 39.9 to 41.1 years (*p* < 0.001).

By combining first and second diagnosis, a total of 3,138,689 psychiatric diagnoses were registered. The majority of the clients was diagnosed with mood disorders (53.9%), primarily depressive episodes (26.7%) and recurrent depressive disorders (24.7%), followed by neurotic, stress-related and somatoform disorders (43.9%), particularly anxiety (18.8%) as well as adjustment and stress-related disorders (13.0%), and substance use disorders (15.3%), mostly alcohol use disorders (8.2%). This order is also valid when considering the first diagnosis only. For the second diagnosis, the most frequent psychiatric diagnoses comprised neurotic, stress-related and somatoform disorders, followed by mood disorders. These top three diagnoses accounted for about 80% of all diagnoses (first: 81.2%, second: 79.4%). The prevalence rates of all diagnoses are presented in Table 1.

**Table 1:** Prevalence rates of mental and behavioral disorders according to ICD-10 diagnostic criteria

Field of diagnosis	Total		First diagnosis		Second diagnosis	
	%	*N*	%	*N*	%	*N*
F00-F09: Organic, including symptomatic, mental disorders	1.2	25,663	0.6	12,313	1.0	14,202
F10-F19: Mental and behavioral disorders due to psychoactive substance use	15.3	339,644	10.5	202,835	13.3	189,026
F10: Alcohol	8.2	181,976	5.8	113,150	5.3	75,297
F11: Opioids	1.0	22,994	0.8	15,774	0.5	7502
F12: Cannabinoids	2.7	59,038	0.9	17,775	3.0	42,579
F19: Multiple drug use and use of other psychoactive substances	3.4	75,379	2.5	47,534	2.1	30,113
F13-F18: Other psychoactive substances	1.9	41,799	0.5	8602	2.4	33,535
F20-F29: Schizophrenia, schizotypal and delusional disorders	5.8	128,125	5.9	113,411	1.5	21,402
F20: Schizophrenia	3.9	86,790	4.1	79,094	0.8	11,564
F21-F29: Other nonorganic psychotic disorders	2.0	44,027	1.8	34,317	0.7	9838
F30-F39: Mood [affective] disorders	53.9	1,193,570	45.3	879,598	24.3	346,380
F31: Bipolar affective disorder	1.3	29,345	1.3	24,496	0.4	5085
F32: Depressive episode	26.7	590,302	21.5	416,531	12.4	175,903
F33: Recurrent depressive disorder	24.7	546,882	21.4	415,651	9.3	132,496
F34: Persistent mood [affective] disorders	2.4	52,435	1.1	21,173	2.2	31,344
F30, F38-F39: Other mood [affective] disorders	0.2	3291	0.0	1747	0.0	1552
F40-F48: Neurotic, stress-related and somatoform disorders	43.9	971,777	25.4	493,605	41.8	595,316
F40: Phobic anxiety disorders	5.3	117,907	2.4	46,986	5.2	73,885
F41: Other anxiety disorders	13.5	298,524	7.3	142,282	11.9	169,112
F42: Obsessive-compulsive disorder	1.4	30,318	0.6	12,261	1.3	18,245
F43: Reaction to severe stress, and adjustment disorders	13.0	288,471	7.5	144,833	10.4	147,399
F45: Somatoform disorders	12.5	277,692	6.4	124,010	11.0	156,614
F44, F48: Other neurotic disorders	2.4	52,957	1.2	23,233	2.0	30,061
F50-F59: Behavioral syndromes associated with physiological disturbances and physical factors	1.5	32,758	0.5	8924	1.7	24,050
F60-F69: Disorders of adult personality and behavior	10.4	229,439	5.3	102,569	9.2	131,326
F60: Specific personality disorders F60.3: Emotionally unstable personality disorders	7.3 4.4	162,119 96,787	3.9 2.7	74,977 53,056	6.2 3.1	88,761 43,997
F61: Mixed and other personality disorders	1.9	42,728	0.9	17,166	1.8	25,574
F62-F69: Other disorders of adult personality and behavior	1.2	27,266	0.5	10,426	1.2	16,991
F70-F79: Mental retardation	2.5	55,802	1.9	36,851	1.4	20,007
F80-F89: Disorders of psychological development F84: Pervasive developmental disorders	3.5 1.3	77,886 28,997	2.4 1.1	47,035 22,000	2.5 0.5	36,216 7179
F90-F98: Behavioral and emotional disorders with onset usually occurring in childhood and adolescence	3.4	75,907	1.9	36,875	3.1	43,791
F90.0: Attention-deficit hyperactivity disorder	2.0	43,518	1.0	18,933	1.7	24,617
F99: Unspecified mental disorder	0.4	8118	0.3	5878	0.2	2241

The column “total” is given as number and percentage of clients with the respective first or second diagnosis (or both) of all 2,213,048 clients. First diagnosis was available from 1,939,894 and second diagnosis from 1,423,957 clients. Percentages for first and second diagnoses were calculated on the basis of these numbers

The highest mean age among all first psychiatric diagnoses was found in the categories of organic mental disorders (44.6 ± 13.6 years), followed by mood disorders (43.6 ± 12.5 years), neurotic, stress-related and somatoform disorders (41.6 ± 12.9 years) and substance use disorders (41.0 ± 11.6 years). On the other hand, the youngest clients were identified among the categories of developmental disorders (21.4 ± 8.7 years), behavioral and emotional disorders with onset usually occurring in childhood and adolescence (22.2 ± 9.7 years), mental retardation (24.7 ± 11.6 years) and personality disorders (30.9 ± 11.4 years). In terms of gender, the diagnostic categories of substance use disorders (78.9%), behavioral and emotional disorders with onset usually occurring in childhood and adolescence (71.9%) and development disorders (69.9%) had the highest proportion of males, whereas clients diagnosed with behavioral syndromes associated with physiological disturbances and physical factors (77.3%), neurotic, stress-related and somatoform disorders (60.3%) and mood disorders (58.5%) were predominantly female. The mean age and sex distribution per diagnostic category are given in Table 2.

**Table 2:** Mean age and sex distribution according to mental and behavioral disorders as first diagnosis

First Diagnosis	Mean age (SD)	Male sex (%, *N*)
F00-F09: Organic, including symptomatic, mental disorders	44.6 (13.6)	65.3 8036
F10-F19: Mental and behavioral disorders due to psychoactive substance use	41.0 (11.6)	78.9 160,028
F20-F29: Schizophrenia, schizotypal and delusional disorders	33.9 (11.2)	65.5 74,296
F30-F39: Mood [affective] disorders	43.6 (12.5)	41.5 365,176
F40-F48: Neurotic, stress-related and somatoform disorders	41.6 (12.9)	39.7 196,024
F50-F59: Behavioral syndromes associated with physiological disturbances and physical factors	32.7 (12.5)	22.7 2030
F60-F69: Disorders of adult personality and behavior	30.9 (11.4)	41.6 42,704
F70-F79: Mental retardation	24.7 (11.6)	58.3 21,502
F80-F89: Disorders of psychological development	21.4 (8.7)	69.6 32,755
F90-F98: Behavioral and emotional disorders with onset usually occurring in childhood and adolescence	22.2 (9.7)	71.9 26,526
F99-F99: Unspecified mental disorder	37.1 (13.4)	49.2 2890

SD = standard deviation

With regard to the proportion of mental disorders within different age groups, mood disorders showed a continuous increase from 15.9% in the younger age group (15–19 years) up to 61.7% in the elder age group (60–69 years). Similarly, the proportion of neurotic, stress-related and somatoform disorders increased from 15.9 to 27.5% across the same age groups. Substance use disorders had the highest proportion in the middle age groups (30–49 years; 13.0-13.4%) with a subsequent continuous decrease to 6.4% in the elder age group (60–69 years). Schizophrenia, personality disorders, mental retardation, developmental disorders as well as behavioral and emotional disorders with onset usually occurring in childhood and adolescence were most frequent up to the age of 29 years and rather rare among elder clients. The proportions of psychiatric diagnoses within the different age groups are presented in Table 3.

**Table 3:** Prevalence rates of mental and behavioral disorders as first diagnosis according to age groups

First diagnosis	Age groups (years)					
	15–19 (*N* = 90189)	20–29 (*N* = 351831)	30–39 (*N* = 357900)	40–49 (*N* = 409825)	50–59 (*N* = 559761)	60–69 (*N* = 170134)
	%, *N*	%, *N*	%, *N*	%, *N*	%, *N*	%, *N*
F00-F09: Organic, including symptomatic, mental disorders	0.4 378	0.5 1675	0.5 1619	0.5 2037	0.8 4447	1.3 2154
F10-F19: Mental and behavioral disorders due to psychoactive substance use	3.4 3081	7.8 27,561	13.4 47,861	13.0 53,360	10.7 60,137	6.4 10,834
F20-F29: Schizophrenia, schizotypal and delusional disorders	3.5 3189	10.1 35,365	10.0 35,933	5.4 22,011	2.6 14,406	1.5 2503
F30-F39: Mood [affective] disorders	15.9 14,361	32.4 114,015	39.2 140,250	48.0 196,529	55.3 309,450	61.7 104,972
F40-F48: Neurotic, stress-related and somatoform disorders	15.9 14,323	22.2 77,959	24.9 88,966	27.4 112,407	27.4 153,166	27.5 46,763
F50-F59: Behavioral syndromes associated with physiological disturbances and physical factors	0.6 566	0.9 3299	0.6 2148	0.3 1345	0.2 1292	0.2 274
F60-F69: Disorders of adult personality and behavior	6.0 5398	12.6 44,205	7.4 26,355	3.5 14,409	1.9 10,624	0.9 1577
F70-F79: Mental retardation	14.0 12,622	4.0 14,036	1.3 4507	0.7 2725	0.5 2580	0.2 364
F80-F89: Disorders of psychological development	22.1 19,970	5.4 19,111	1.3 4813	0.4 1699	0.2 1192	0.1 168
F90-F98: Behavioral and emotional disorders with onset usually occurring in childhood and adolescence	17.7 15,957	3.7 13,184	1.2 4247	0.5 1992	0.2 1238	0.1 153
F99-F99: Unspecified mental disorder	0.4 344	0.4 1421	0.3 1201	0.3 1311	0.2 1229	0.2 372

The percentages are shown column-wise per age group. The total number of assessed clients amounts to *N* = 1,939,640, because age of the client was not reported in 254 cases

Across the time period between 2016 and 2021, the prevalence rates of first and second diagnoses showed some changes which are shown in Table 4. In particular, the prevalence rates of mood disorders as well as neurotic, stress-related and somatoform disorders continuously increased from 51.3 to 55.7% and from 40.4 to 46.3%, respectively. At the same time, substance use disorders and personality disorders continuously dropped from 16.3 to 13.9% and from 11.2 to 9.4%, respectively. All other diagnoses did not markedly change over time.

**Table 4:** Prevalence rates of mental and behavioral disorders (first and secondary diagnoses), temporal development from 2016 to 2021

Field of diagnosis	First and secondary diagnoses					
	2016 (*N* = 279427)	2017 (*N* = 323343)	2018 (*N* = 353082)	2019 (*N* = 439727)	2020 (*N* = 423391)	2021 (*N* = 394078)
	%, *N*	%, *N*	%, *N*	%, *N*	%, *N*	%, *N*
F00-F09: Organic, including symptomatic, mental disorders	1.1 3145	1.1 3550	1.2 4202	1.1 4958	1.2 4918	1.2 4890
F10-F19: Mental and behavioral disorders due to psychoactive substance use	16.3 45,685	16.1 51,929	15.7 55,414	16.0 70,144	14.5 61,583	13.9 54,889
F20-F29: Schizophrenia, schizotypal and delusional disorders	6.1 17,158	6.0 19,454	6.0 21,221	5.6 24,685	5.6 23,544	5.6 22,063
F30-F39: Mood [affective] disorders	51.3 143,475	53.0 171,224	54.0 190,572	53.7 236,281	54.9 232,509	55.7 219,509
F40-F48: Neurotic, stress-related and somatoform disorders	40.4 112,848	42.3 136,826	43.4 153,185	43.9 193,223	45.6 193,163	46.3 182,532
F50-F59: Behavioral syndromes associated with physiological disturbances and physical factors	1.4 4045	1.5 4970	1.5 5208	1.4 6163	1.5 6561	1.5 5811
F60-F69: Disorders of adult personality and behavior	11.2 31,418	11.1 36,052	10.9 38,404	10.3 45,488	9.7 41,136	9.4 36,941
F70-F79: Mental retardation	2.7 7600	2.6 8471	2.5 8927	2.4 10,571	2.4 10,276	2.5 9957
F80-F89: Disorders of psychological development	3.6 10,125	3.5 11,434	3.5 12,428	3.4 14,879	3.4 14,281	3.7 14,739
F90-F98: Behavioral and emotional disorders with onset usually occurring in childhood and adolescence	3.4 9520	3.5 11,315	3.4 12,030	3.3 14,459	3.4 14,518	3.6 14,065
F99-F99: Unspecified mental disorder	0.4 1041	0.4 1443	0.4 1569	0.4 1753	0.3 1302	0.3 1010

Note: The percentages were calculated column by column for each year and show the percentage of the specific diagnosis group of all assessed clients in the respective year. Since a client may have one or two psychiatric diagnoses, the percentages do not add up to 100%, but higher

Considering the work ability, 19.0% (*N* = 419,581) of the socio-medical assessments did not provide any specification and were excluded from further analysis. Of the remaining population, 53.4% of the clients with a first and/or second psychiatric diagnosis were currently not able to work (less than 3 h per day), 6.2% were assessed to be able to work part time (between 3 and 6 h per day), and 40.4% were able to work full time (six or more hours per day). Clients with a first diagnosis of organic mental disorders (74.9%), schizophrenia (74.4%) and mental retardation (80.3%) were most likely to be evaluated as not being able to work at all, followed by substance use disorders (61.1%), personality disorders (56.6%) and mood disorders (54.2%). Otherwise, a relatively large proportion of clients with a diagnosis of behavioral and emotional disorders with onset usually occurring in childhood and adolescence (58.4%) and developmental disorders (51.0%), but also neurotic, stress-related and somatoform disorders (44.0%) and mood disorders (40.1%) were assessed to be able to work full time. Similar patterns were obtained for mental disorders as second diagnosis. The respective distribution of the degree of work ability depending on the psychiatric diagnoses is given in Table 5.

**Table 5:** Work ability according to first and second diagnosis

	First diagnosis			Second diagnosis		
	< 3 h	3–6 h	> 6 h	< 3 h	3–6 h	> 6 h
	%, *N*	%, *N*	%, *N*	%, *N*	%, *N*	%, *N*
F00-F09: Organic, including symptomatic, mental disorders	74.9 7775	5.7 597	19.4 2015	68.8 8266	6.1 729	25.2 3025
F10-F19: Mental and behavioral disorders due to psychoactive substance use	61.1 98,353	6.9 11,113	32.0 51,592	58.3 90,489	6.2 9618	35.5 55,132
F20-F29: Schizophrenia, schizotypal and delusional disorders	74.4 71,303	6.4 6127	19.2 18,415	72.8 13,172	6.0 1089	21.2 3844
F30-F39: Mood [affective] disorders	54.2 390,948	5.7 41,367	40.1 288,827	52.7 148,835	6.5 18,309	40.8 115,073
F40-F48: Neurotic, stress-related and somatoform disorders	49.1 195,034	7.0 27,677	44.0 174,658	55.0 269,508	6.1 29,900	38.9 190,748
F50-F59: Behavioral syndromes associated with physiological disturbances and physical factors	54.9 4039	5.9 434	39.2 2883	53.8 10,833	6.5 1318	39.7 7987
F60-F69: Disorders of adult personality and behavior	56.6 47,502	7.4 6226	36.0 30,224	57.5 62,822	6.6 7232	35.9 39,219
F70-F79: Mental retardation	80.3 24,474	2.2 674	17.5 5320	74.4 12,018	3.3 530	22.3 3599
F80-F89: Disorders of psychological development	46.1 17,598	2.9 1116	51.0 19,485	50.1 14,940	3.2 960	46.7 13,945
F90-F98: Behavioral and emotional disorders with onset usually occurring in childhood and adolescence	37.2 10,991	4.4 1299	58.4 17,260	46.4 16,749	4.6 1650	49.0 17,665
F99-F99: Unspecified mental disorder	72.4 3188	4.6 201	23.0 1015	63.0 1117	6.4 113	30.6 542

Note: The percentages were calculated row by row for each diagnosis group and show the proportion of clients considered to be able to work < 3 h, 3–6 h, and > 6 h per day in relation to the first and second diagnosis

## Discussion

Job loss and unemployment are amongst the most stressful life events and have detrimental effects on mental health [8], and, conversely, poor mental health significantly increases the risk of unemployment [2]. This analysis explored the prevalence of mental disorders as well as the work ability among unemployed individuals in Germany who underwent socio-medical assessment by the FEA between 2016 and 2021. Socio-medical assessments are only carried out on request of FEA counselors if physical or mental health issues which might affect a person’s work ability are assumed [22]. Therefore, the findings of this analysis cannot be transferred to all unemployed people, although they are valid for a proportion of 500,000 unemployed people per year.

About 52% of the FEA clients who underwent socio-medical assessment were diagnosed with at least one mental disorder. As only the two most relevant diagnoses were registered in the FEA database, it cannot be excluded that the real prevalence rate of mental disorders was even higher. Of note, about 80% of all psychiatric diagnoses can be attributed to only three diagnostic categories, i.e., mood disorders (39.1%), neurotic, stress-related and somatoform disorders (34.7%) and substance use disorders (12.5%). Mood disorders and neurotic, stress-related and somatoform disorders were more common in female individuals whereas clients with substance use disorders were predominantly male. The prevalence rates in this sample are markedly higher than in the general population in Germany for which twelve-month prevalence rates of 9.3% for mood disorders, 15.3% for anxiety disorders and 5.7% for substance use disorders were reported [23].

The results of this study are largely in line with international as well as national data. A recent systematic review presented the prevalence rates of several mental disorders among unemployed people in different geographic regions [11]. In terms of Europe and America, mean prevalence rates of 32.5% and 51.9% for mood disorders, 12.4% and 15.4% for anxiety disorders, and 26.0% and 19.2% for other psychiatric diagnoses (including substance use disorders), respectively, were reported. Two recent studies from Germany which were carried out in two different local job centers reported prevalence rates among long-term unemployed people for mood disorders of 60.5% and 60.7%, for neurotic, stress-related and somatoform disorders of 58.1% and 42.5%, and for substance use disorders of 21.5% and 20.9%, respectively [24–25]. The mean age and sex distribution were similar to the present study. The lower prevalence rates of this analysis might be explained by the restriction to only two diagnoses per case and the consideration of nationwide data, whereas the other two studies included all psychiatric diagnoses available in their databases and assessed unemployed people exclusively from their local (urban) catchment areas. In this context, urban environments have been found to be associated with higher prevalence rates of mental disorders compared to rural areas [23]. Moreover, the two aforementioned studies exclusively focused on long-term unemployment that has been related to an even higher burden of mental illness [26], whereas this study included long-term as well as short-term unemployed.

It seems to be obvious that the relatively high prevalence rates for mood disorders, mainly depressive episodes, as well as neurotic, stress-related and somatoform disorders, especially generalized anxiety and panic disorders, among unemployed individuals can be linked to psychological distress due to loss of income and financial security, deprivation of the latent functions of employment, with collective purpose identified as the most important latent function, and stigmatization [16, 27]. On the other hand, substance use disorders, particularly alcohol abuse, may develop as a dysfunctional strategy to cope with symptoms of psychological distress, depression and anxiety [19]. However, the methodological limitations of this analysis do not allow to differentiate whether the mental disorders were already present before job loss or subsequently developed during unemployment.

Interestingly, the prevalence rates of some psychiatric diagnoses among unemployed people changed across the time period between 2016 and 2021, although the rates of mental disorders in the general population usually remain relatively stable over time [28]. In particular, there was a marked increase in the prevalence of mood disorders by about 8.6% and of neurotic, stress-related and somatoform disorders by about 14.6%.

In this context, it is noteworthy that the time period of this evaluation included the COVID-19 pandemic which had a significant impact on mental health. The pandemic reached Germany in the beginning of 2020 and resulted in two strict lockdowns in 2020 and 2021 [29]. It can be speculated whether the increased prevalence rates were due to the societal and health-related consequences of the pandemic. In fact, several epidemiological studies reported an initial increase of depression and anxiety symptoms in the general population, particularly in the first two months of the pandemic [30]. After a peak in April and May 2020, however, depression and anxiety symptoms decreased again although the prevalence of mental health problems remained high during the entire pandemic [31]. It therefore remains unclear to what degree the changes in the prevalence of depression and anxiety among unemployed individuals might have been driven by the pandemic. Similarly, the decreased prevalence of substance (alcohol) use disorders by about 14.7% cannot be fully explained by the pandemic. According to several observational studies, more people reduced their alcohol use since the onset of the pandemic, but high-risk alcohol users rather increased their drinking levels [32].

In those people who became unemployed during the observation period of this analysis, distinct work-related factors may have most probably contributed to the increase of the prevalence rates of depression and anxiety disorders. Within the last decade, job demands have rapidly increased due to innovation, digitalization and demographic transition, often resulting in work overload, dissolution of temporal and spatial boundaries, low job control, work discontent, and chronic stress. These consequences may in turn contribute to the development of depression and anxiety and may increase the risk for job loss and unemployment [33–35].

Mental disorders strongly affect the work ability depending on the variety, intensity, and duration of their specific symptoms which, in turn, may result in reduced functions and impaired capacities [36]. In this study, 53.4% of the unemployed clients with at least one psychiatric diagnosis were currently not able to work (less than 3 h per day), according to the results of the socio-medical assessments. The three most prevalent mental disorders could be identified to be primarily responsible for this result, with a rate of full inability to work between 50 and 60%.

Despite the high prevalence rates of mental disorders, recent studies from Germany showed that the utilization of mental health care was found to be disproportionately low: Only less than half of the unemployed people with mental disorders were in current psychiatric and/or psychotherapeutic treatment, and only slightly more than 50% of the pharmacotherapies, partly prescribed by general practitioners, were in accordance with the national treatment guidelines [24–25]. Collaboration programs between job centers and mental health care institutions as well as low-threshold access to mental health care may be beneficial in order to prevent further chronification of mental disorders and, thereby, improve employment perspectives.

On the other hand, more than 40% of all clients were assessed to be able to work full time (six or more hours per day), particularly those with mood disorders (40.1%) as well as neurotic, stress-related and somatoform disorders (44.0%). In this respect, a recent study from Germany found that about two-thirds of unemployed people with a severe mental illness expressed a strong desire to work in the general labor market [4]. With regard to the present study, this finding indicates that especially the subgroup of unemployed individuals with depression or anxiety who were evaluated to be fully able to work may benefit from even more specific and individualized support and supported employment initiatives beyond those that already exist. They have been recommended as the most effective psychosocial intervention for people with severe mental disorders, regardless of diagnostic, clinical, functional and personal characteristics, showing significant effects on employment rates and job retention, but also on psychopathology and quality of life, besides traditional vocational rehabilitation programs [37–38]. Supported employment interventions include not only placement in competitive employment, systematic training on the job and long-term support by job coaching but also integrated mental health care.

Other diagnoses, such as organic mental disorders, schizophrenia, mental retardation and disorders of psychological development including autism spectrum disorders, were rather rare, compared to the prevalence rates for other psychiatric diagnoses, and mostly associated with full inability to work. It can be assumed that people who were affected by one of these severe and persistent mental disorders were already excluded from the labor market at earlier age, participated in prevocational training programs or worked in sheltered employment [38].

This study has some limitations. Firstly, only unemployed individuals who underwent socio-medical assessment by the FEA were included in the analysis. This methodological approach does not allow to draw any conclusions about the mental health status of the entire population of unemployed people in Germany. Secondly, the database only provides information about the two main diagnoses and further diagnoses are not available. It can therefore be assumed that the prevalence rates of mental disorders were even higher. Thirdly, the socio-medical assessments were not based on standardized interviews. Therefore, it cannot be excluded that the findings were influenced by, for example, the clinicians’ subjective diagnostic preferences or current trends in diagnostics. Fourthly, 3.5% of all subjects did not have any diagnosis in the database. Some persons failed to appear to the socio-medical assessment, while others were assessed at the same time by other federal authorities such as the German Pension Insurance with pending decision. On the other hand, it cannot be ruled out that some individuals were included more than once. However, the likelihood of multiple socio-medical assessments of the same person is rather low given the relatively short time period of six years. Fifthly, work ability was not determined in 19% of the cases. The documentation of the work ability is not mandatory and reasons for not completing may be uncertainty about the result or documentation in free text fields. And finally, the database lacks additional sociodemographic, health-related and occupational data about the unemployed persons due to data protection regulations. In particular, detailed information about social background, education, occupation and unemployment, mental health as well as former and current treatments is missing. Based on the available data, it is also not possible to distinguish between short- and long-term unemployed and to draw conclusions about the progress, interactions and outcomes of unemployment and mental disorder.

Despite these limitations, this study also has strengths. Firstly, this analysis includes the large number of all unemployed people in Germany who underwent socio-medical assessment, and the entire spectrum of diagnostic categories, covering a time period of six years. Secondly, the results are based on direct evaluations by medical practitioners who are specialized on social medicine and, in the uppermost cases, had access to the comprehensive medical history of the respective clients; in contrast to the majority of other studies that mainly applied a census approach or used data from national surveys [11]. And thirdly, the database did not only inform about the two main diagnoses but also provided information about the work ability in relation to distinct mental disorders.

## Conclusions

This study revealed a high prevalence of mental disorders of about 52% among all unemployed individuals who underwent socio-medical assessment by the FEA. Mood disorders, neurotic, stress-related and somatoform disorders, and substance use disorders showed the highest prevalence rates and accounted for about 80% of all psychiatric diagnoses. However, about 40% of them were evaluated to be able to work full time. From the public health perspective, close collaborations between unemployment agencies, job centers and mental health care institutions as well as specific and individualized interventions are required in order to improve mental health status, prevent further chronification of mental disorders, avoid labor market exit, and promote return to competitive employment.

## Data Availability

The data that support the findings of this study are available from the Medical Service of the Federal Employment Agency but restrictions apply to the availability of these data, which were used under license for the current study, and so are not publicly available. Data are however available from the authors upon reasonable request and with permission of the Medical Service of the Federal Employment Agency.
